# *Neurogenin-3* Enteric Endocrinopathy: A Rare Case of Pediatric Congenital Diarrhea and Diabetes Mellitus

**DOI:** 10.1097/PG9.0000000000000173

**Published:** 2022-02-04

**Authors:** Kendra L. Francis, Arushi Verma, M. Cristina Pacheco, Danielle Wendel, Padade M. Vue, Shannon J. Hu, Jarrad M. Scarlett

**Affiliations:** From the * Department of Pediatric Gastroenterology and Hepatology, Seattle Children’s Hospital, Seattle, WA; † University of Washington Medicine Diabetes Institute, Department of Medicine, Seattle, WA; ‡ Department of Pediatric Endocrinology, University of Nevada Reno School of Medicine, Reno, NV; § Department of Laboratories, Seattle Children’s Hospital, Seattle, WA; ∥ Department of Pathology and Laboratory Medicine, University of Washington, Seattle, WA.

**Keywords:** enteroendocrine cell, transcription factor, intestine, pancreas, intestinal failure

## Abstract

Disorders of intestinal enteroendocrine cells (EEC) are a rare cause of congenital diarrhea and diabetes. The gene *NEUROG3* is essential in EEC differentiation, and mutations in this gene lead to a paucity of EEC in the intestine and pancreas, often presenting clinically as congenital diarrhea and diabetes mellitus. We present the earliest known diagnosis of *NEUROG3*-associated enteric endocrinopathy, which was identified on a neonatal diabetes genetic panel sent at 4 weeks of age. Our patient presented with severe diarrhea, malnutrition, electrolyte derangements, and neonatal diabetes. He was started on parenteral nutrition at 3 months of age for nutritional and hydration support and required long-acting insulin for his diabetes. We demonstrate significant reduction in EEC, including cells expressing glucagon-like peptide-1, in intestinal biopsies from our patient, raising the possibility that loss of glucagon-like peptide-1 contributes to *NEUROG3*-associated diarrhea and diabetes mellitus. This case advances our understanding of the presentation, diagnosis, and management of this rare disease.

## INTRODUCTION

Congenital diarrheal disorders (CDD) are a group of inherited enteropathies that most commonly present within the first weeks of life with severe, life-threatening diarrhea and malabsorption of nutrients. The etiology of CDD is diverse and includes defects in electrolyte transport, impaired digestive enzyme function, improper enterocyte trafficking and polarity, immune dysregulation, and defects of enteroendocrine cell (EEC) differentiation and function.^1,2^ One rare CDD characterized by malabsorptive diarrhea and paucity of intestinal EEC, termed “enteric anendocrinosis,” is caused by disruption of EEC differentiation due to loss-of-function mutations in the *Neurogenin-3* (*NEUROG3*) gene. We present the earliest described case diagnosed with *NEUROG3*-associated enteric endocrinopathy, highlighting the critical role these cells play in intestinal nutrient absorption.

Neurogenin-3 (NEUROG3, encoded by *NEUROG3*) is a basic helix-loop-helix transcription factor located on chromosome 10q21-22 that is expressed in endocrine progenitor cells and drives endocrine cell fate in the intestine and pancreas. Homozygous mutations in the *NEUROG3* gene disrupt the cell differentiation pathways of enteric stem cells and pancreatic islets, causing a significant reduction to complete lack of endocrine cells in the intestine and pancreas.^3,4^

Ten patients with homozygous NEUROG3 mutations have been reported previously. Each patient suffered from severe congenital malabsorptive diarrhea necessitating parenteral nutrition (PN).^5–11^ In contrast, the presence of neonatal diabetes and exocrine pancreatic deficiency in patients was variable.^5–11^ We present a case of a male patient with insulin-dependent diabetes and severe diarrhea from birth found to have a homozygous *NEUROG3* mutation at 4 weeks of age.

## CASE

Our patient was born small for gestational age (1.69kg) at 37 weeks and was noted to have periodic non-ketotic hyperglycemia up to 270 mg/dL and non-bloody diarrhea since day of life 1. Newborn screens were normal. He remained in the neonatal intensive care unit for the first 3 weeks of life for glucose monitoring and fluid support. After trialing breastmilk and partially hydrolyzed formula, he had mild improvement in stool output with 22 kcal/oz extensively hydrolyzed formula, which he was discharged on at 3 weeks of age feeding *ab lib* by mouth.

He was readmitted to the hospital at 4 weeks of age for hyperglycemia and metabolic acidosis, at which time a neonatal diabetes genetic panel consisting of 23 genes associated with monogenic diabetes was sent (Neonatal Diabetes Gene Panel, Seattle Children’s Hospital Molecular Genetics Laboratory). At 7 weeks of age, results of the genetic panel revealed a homozygous p.I132F c.394A>T novel variant in the *NEUROG3* gene, which was deemed likely pathogenic. During this admission, he gained weight appropriately on the same extensively hydrolyzed formula *ad lib* with persistent diarrhea and stable electrolytes. Additionally, his pre-prandial glucose levels were <200 mg/dL. He was discharged with home glucose monitoring and outpatient endocrinology and gastroenterology clinic follow up.

The patient was then re-admitted at 3 months of age with dehydration, acidosis, and electrolyte derangements with acute on chronic diarrhea (Table 1). His weight on admission was 3.29 kg with a z-score of −5.77, and weight-for-length was <third percentile. Electrolytes improved after several intravenous (IV) fluid boluses, but when IV fluids were held and he was allowed oral intake, he developed recurrent metabolic acidosis. Additional stool studies included a normal fecal elastase, normal stool alpha-1 antitrypsin, increased fecal fatty acids, and calculated stool anion gap of 128 mOsm/kg (Table 1). Vitamin E and Vitamin D levels were low, and Prothrombin Time (16.9 seconds) was prolonged, indicating significant fat-soluble vitamin deficiencies (Table 1). He was started on supplementation for Vitamins A, D, E, and K. A small bowel follow-through fluoroscopic study showed normal small bowel transit time.

**TABLE 1.** Laboratory and Diagnostic Workup Leading up to PN Initiation at 3 Months of Age and at 6 Months of Age While on PN

Laboratory or diagnostic test	Result at 3 months of age	Result at 6 months of age
Serum sodium	153 mEq/L	136 mEq/L
Serum chloride	128 mEq/L	103 mEq/L
Serum bicarbonate	13 mEq/L	23 mEq/L
Blood glucose	115 mg/dL	121 mg/dL
Vitamin E level	3.5 mg/L	11.3 mg/L
Vitamin D level	10 ng/mL	15.2 ng/mL
Prothrombin time	16.9 seconds	15.2 seconds
Weight percentile	<0.01 percentile	15.99 percentile
Fecal elastase	38 μg/g	—
Stool α1 anti-trypsin	<0.05 mg/g	—
Fecal fatty acids	>100 droplets/HPF	—
Stool anion gap	128 mOsm/kg	—

Workup revealed a hypernatremic, hyperchloremic metabolic acidosis and low fat-soluble vitamin levels, all of which improved after PN initiation. Weight percentile increased after 3 months on PN. Stool studies at diagnosis were normal except for elevated fecal fatty acids and elevated stool anion gap.

PN = parenteral nutrition.

**FIGURE 1.**
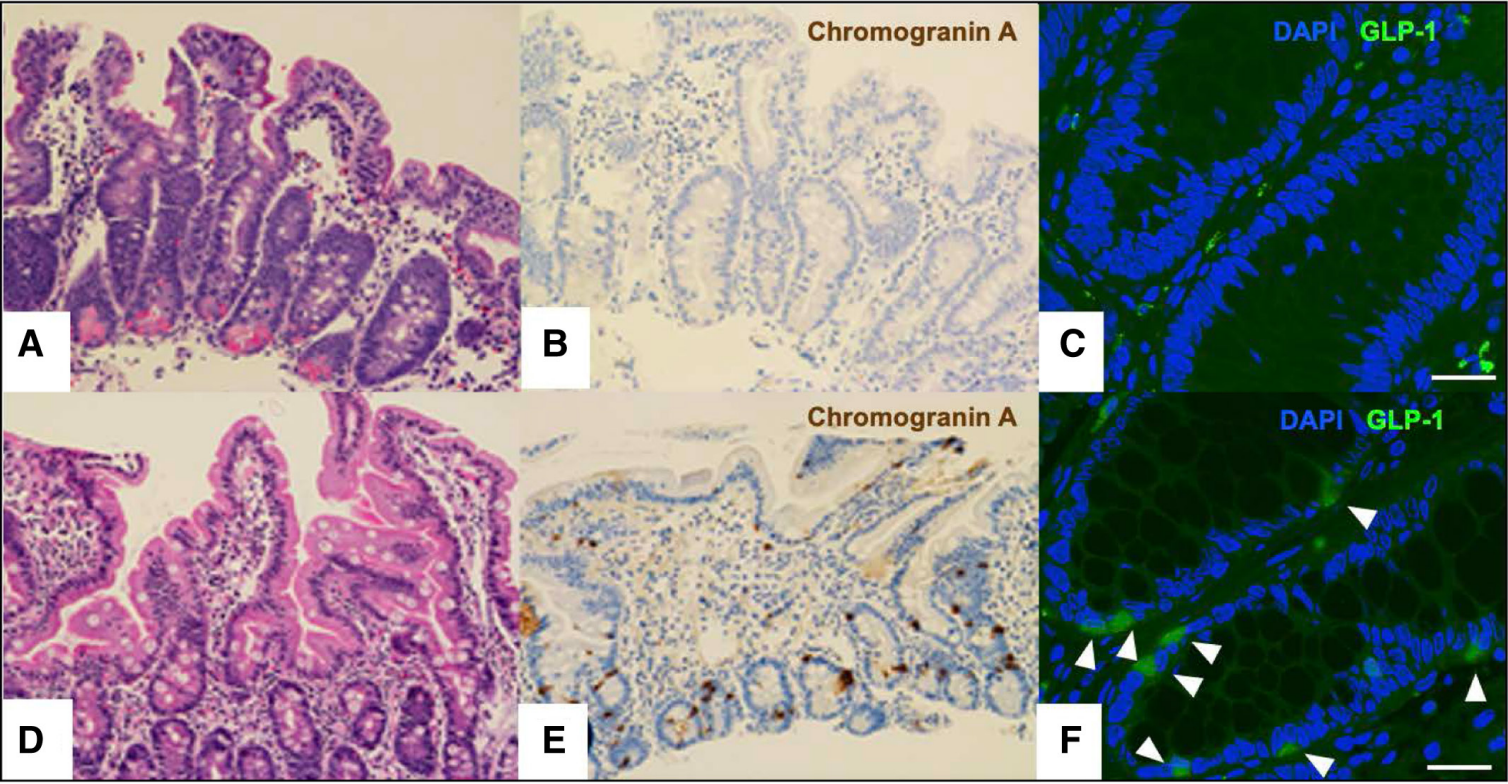
Intestinal biopsies demonstrate a paucity of enteroendocrine cells as stained using Chromogranin-A and GLP-1. Patient (A) duodenum with H&E stain (200×), (B) duodenum with Chromogranin A stain (200×), and (C) colon with GLP-1 immunofluorescent stain (scale bar 25 μm). Age-matched control (D) duodenum with H&E stain (200×), (E) duodenum with Chromogranin A stain (200×), and (F) colon with GLP-1 immunofluorescent stain highlighting GLP-1 expressing enteroendocrine cells (arrowheads, scale bar 25 μm).

Esophagogastroduodenoscopy and flexible sigmoidoscopy performed at 3 months of age were macroscopically normal. Pathology revealed a normal brush border in the duodenum and normal colonic architecture (Fig. 1A). Staining for Chromogranin-A, a neuroendocrine marker,^12^ was used to identify EEC, which were rare in the duodenum and absent in the sigmoid colon (Fig. 1B). Glucagon-like peptide (GLP)-1 immunofluorescent staining (Santa Cruz Biotechnology, sc-514592) demonstrated a paucity of GLP-1 staining in the colon compared to age-matched control (Fig. 1C).

The patient’s profuse diarrhea persisted, producing 100 to 200 mL/kg/day of mixed output. Trials to wean off IV fluids were unsuccessful due to recurrent hyperchloremic metabolic acidosis and failure to gain weight on exclusive oral feeding. Therefore, he was started on PN at 3 months of age. On PN providing 100 kcal/kg/day and 110 mL/kg/day without restriction in oral intake, he began to gain weight with sustained growth, resolution of acidosis, and ability to maintain hydration, but had worsening hyperglycemia. While on PN, plasma glucose levels were persistently above 200 mg/dL without ketosis, necessitating subcutaneous insulin.

The patient was discharged at 4 months of age on home PN and unrestricted oral feedings. With sustained growth and normal electrolytes, PN was gradually weaned over the next 6 months and discontinued at 10 months of age. At the time of our writing, the patient is 17 months of age and receives all his nutrition by mouth with a regular diet for age and partially hydrolyzed formula. He was weaned off insulin around 9 months of age.

## DISCUSSION

EEC comprise about 1% of the total epithelium in the intestinal tract, but their role in nutrient absorption and glucose homeostasis is critical for normal growth and survival. Recently, patients with *NEUROG3* mutations have been described as presenting with congenital diarrhea and diabetes. These patients lack EEC within the intestine, resulting in enteric anendocrinosis.^8^ The 10 reported cases with homozygous mutations in the *NEUROG3* gene all developed intestinal failure (IF) from birth or within the first few weeks of life, supporting a key role for EEC in regulating enterocyte function. Our patient has a novel mutation in the *NEUROG3* gene, a homozygous p.I132F missense change not previously reported.

Intestinal EEC are classified based on hormone production and/or distribution in the gastrointestinal tract; at least 15 different types have been characterized.^13^ In addition to the hormones that EEC secrete to regulate digestive processes and glucose homeostasis, EEC are also directly involved in sensing intraluminal pH and osmotic load.^8^ Homozygous mutations in the *NEUROG3* gene in humans and mice result in complete or near-complete absence of EEC in the small and large intestine,^14^ confirming that expression of the NEUROG3 transcription factor is required to induce intestinal endocrine progenitor cells. Paucity of EEC in the intestine was confirmed by our patient’s lack or near-complete lack of Chromogranin-A staining in intestinal biopsies from both the duodenum and colon and decreased GLP-1 staining in the colon (Fig. 1). GLP-1 induces expansion of insulin-secreting β-cell mass, augments glucose-stimulated insulin secretion, and is an important mediator of “ileal brake” activation, which influences intestinal transit and optimizes absorption.^15^ It is produced in intestinal EEC alongside GLP-2, which also influences intestinal transit and villous growth.^16^ Although the cause of diabetes mellitus, malabsorptive diarrhea and poor growth in patients with *NEUROG3* mutations is likely multifactorial, deficiency of hormones produced by EEC including GLP-1 and GLP-2 may play a critical role in the underlying pathophysiology. In this context, it is notable that both GLP-1 and GLP-2 analogs exist as treatments for other clinical conditions and may benefit patients with *NEUROG3* mutations, though the necessary studies have yet to be conducted and numbers would be limited by the rarity of this disease.

Although disorders of EEC are rare, they represent an important cause of IF. The pathology resulting from mutations involving EEC in the intestine highlights the role of these cells in digestion, absorption, and glucose homeostasis. Specifically, the *NEUROG3* gene, coding for NEUROG3 transcription factor, plays an integral role in the development of EEC and pancreatic β-cells, and its mutations result in IF and insulin-dependent diabetes mellitus.

## ACKNOWLEDGMENTS

K.L.F., A.V., P.M.V., D.W., and J.M.S. were responsible for the patient during the outpatient and hospital courses, interpreted the findings and were responsible for clinical decision making. K.L.F. and J.M.S. wrote the manuscript. A.V., P.M.V., D.R.W., and M.C.P. critically reviewed the manuscript. M.C.P. performed the histopathological analysis. S.J.H. assisted with immunohistochemical staining and image acquisition. All authors read and approved the final manuscript.
